# Retraction Note: detangling PPI networks to uncover functionally meaningful clusters

**DOI:** 10.1186/s12918-018-0641-3

**Published:** 2018-11-19

**Authors:** Sarah Hall-Swan, Jake Crawford, Rebecca Newman, Lenore J. Cowen

**Affiliations:** Department of Computer Science, Tufts University, Medford, MA 02155 USA

## Retraction note to: BMC Systems Biology 2018, 12(Suppl 3):24 10.1186/s12918-018-0550-5

The authors have retracted this article [1]. After publication they discovered a technical error in the Louvain algorithm with bounded cluster sizes. Correction of this error substantially changed the results for this algorithm and the conclusions drawn in the article were found to be incorrect. The authors will submit a new manuscript for peer review.

All authors agree with this retraction.
